# “Make yourself un-NIMBY-able”: stakeholder perspectives on strategies to mobilize public and political support for overdose prevention centers in the United States of America

**DOI:** 10.1186/s12954-024-00955-6

**Published:** 2024-02-15

**Authors:** Joseph G. Rosen, Erin Thompson, Jessica Tardif, Alexandra B. Collins, Brandon D. L. Marshall, Ju Nyeong Park

**Affiliations:** 1 Department of International Health, Bloomberg School of Public Health, Johns Hopkins University, 615 North Wolfe Street, Room E5031, Baltimore, MD 21205 USA; 2 Harm Reduction Innovation Lab, Rhode Island Hospital, 1125 North Main Street, Providence, RI 02904 USA; 3 Department of Epidemiology, School of Public Health, Brown University, 121 South Main Street, Providence, RI 02903 USA; 4 Department of General Internal Medicine, Warren Alpert Medical School, Brown University, 593 Eddy Street, Providence, RI 02903 USA

**Keywords:** Supervised consumption sites, Opioids, Harm reduction, Injection drug use, People who use drugs, Qualitative research

## Abstract

**Background:**

Overdose prevention centers (OPCs), also known as supervised injection facilities and safe consumption sites, are evidenced-based interventions for preventing overdose deaths and drug-related morbidities. The pathways to legalizing OPCs in the USA have confronted multiple social, political, and legal obstacles. We conducted a multi-site, qualitative study to explore heterogeneities in these pathways in four jurisdictions, as well as to understand stakeholder perspectives on valuable strategies for galvanizing political and public support for OPCs.

**Methods:**

From July 2022 to February 2023, we conducted 17 semi-structured, in-depth interviews with OPC policymakers, service providers, advocates, and researchers from California, New York City, Philadelphia, and Rhode Island, where efforts have been undertaken to authorize OPCs. Using inductive thematic analysis, we identified and compared contextually relevant, salient approaches for increasing support for OPCs.

**Results:**

Participants described several strategies clustering around five distinct domains: (1) embedding OPC advocacy into broader overdose prevention coalitions to shape policy dialogs; (2) building rapport with a plurality of powerbrokers (e.g., lawmakers, health departments, law enforcement) who could amplify the impact of OPC advocacy; (3) emphasizing specific benefits of OPCs to different audiences in different contexts; (4) leveraging relationships with frontline workers (e.g., emergency medicine and substance use treatment providers) to challenge OPC opposition, including ‘NIMBY-ism,’ and misinformation; and (5) prioritizing transparency in OPC decision-making to foster public trust.

**Conclusion:**

While tailored to the specific socio-political context of each locality, multiple OPC advocacy strategies have been deployed to cultivate support for OPCs in the USA. Advocacy strategies that are multi-pronged, leverage partnerships with stakeholders at multiple levels, and tailor communications to different audiences and settings could yield the greatest impact in increasing support for, and diffusing opposition to, future OPC implementation.

## Background

Drug overdoses remain a leading cause of death in the United States of America (USA). Fatal overdoses reached record-shattering levels in 2021, with 106,699 deaths reported in the USA—a 14% increase from the prior year [1]. Since 2016, most fatal overdoses have involved synthetic opioids like fentanyl and, more recently, have been attributed to polysubstance use involving the co-consumption of opioids and psychostimulants [2]. The proliferation of illicitly manufactured fentanyl has dramatically increased the potency of the drug supply, elevating overdose risk for people who use drugs (PWUD). Recent studies from the USA have detected fentanyl and fentanyl analogs with alarming frequency in heroin and stimulant drug samples [3–6], reinforcing the ubiquity and staying power of potent synthetic opioids in illicit drug markets. Addressing surging overdose deaths in the USA, thus, requires innovative solutions beyond the constellation of existing interventions (e.g., naloxone distribution, drug treatment services).

Overdose prevention centers (OPCs)—also known as safe consumption sites, medically supervised injection and inhalation facilities, drug consumption rooms, overdose prevention sites, supervised consumption services, and harm reduction centers—are evidence-based interventions that facilitate rapid response to suspected overdoses and other health emergencies [7–9]. OPCs are spaces where PWUD can bring and use pre-obtained substances under supervision of trained personnel, with some OPCs supporting multiple drug consumption modalities, including inhalation. Operating under medical or peer-based models, OPCs typically offer various onsite services in addition to overdose prevention for clients, including: provision of harm reduction supplies (e.g., sterile injection equipment, naloxone), HIV and hepatitis C testing, healthcare navigation, mental health screening, substance use treatment referrals, and linkage to auxiliary social services like supportive housing [10]. OPCs are also among the most well-studied overdose response interventions globally, with the extant literature attributing significant reductions in overdose deaths [11–13], injection equipment sharing [14], and public drug use [14, 15] to OPC implementation.

Despite the established effectiveness of OPCs globally, as well as their acceptability among PWUD in the USA [16–19], efforts to introduce these sites in the USA have been marred by political and social forces. At present, the legal status of OPCs at a federal level is unclear, but several jurisdictions have formally authorized OPCs through state legislation, or permit their operation at a municipal level, and unsanctioned OPCs have operated throughout the USA for at least a decade [15, 20, 21]. A component of the Anti-Drug Abuse Act, known colloquially as the ‘Crack House Statute,’ federally prohibits leasing, renting, opening, using, or maintaining any space in which illicit substances are manufactured, distributed, or consumed [22]. This statute has been used to challenge efforts to authorize or operate OPCs in the USA, including most recently in a legal challenge by the Department of Justice in attempt to restrict OPC implementation in the city of Philadelphia [23]. At the state level, efforts to circumvent state opposition through alternative legal channels, from emergency decrees to local OPC authorization, have confronted political opposition from elected officials, law enforcement, and organized constituencies [24–26]. Even in the presence of political support, salient barriers to OPC implementation have included resistance from community residents and neighbors (i.e., “Not in my Backyard” or NIMBYism), landlord reticence to rent properties to organizations intending to implement OPCs (fearing perceived liability), and prohibitions on public funds or grants to support OPC operations [25, 27–30]. Collectively, these factors may diminish optimism for the prospect of OPC authorization and implementation in the USA.

OPC advocacy efforts have, however, captured the national spotlight in 2021, following successful municipal OPC implementation in New York City (NYC) and adoption of OPC legislation by the Rhode Island state government. On November 30, 2021, Mayor Bill de Blasio announced the opening of two OPCs in NYC, operated and managed by OnPoint NYC [31, 32]. In the 12 months following the unveiling of OnPoint’s sanctioned OPCs, 650 overdoses were reversed onsite, averting deaths that might have occurred in the absence of supervised drug consumption services [33]. In July 2021, the Rhode Island General Assembly passed OPC legislation, later signed into law by Governor Dan McKee—becoming the first state in the USA to legally authorize a pilot of medically-regulated OPCs (known locally as harm reduction centers), which are anticipated to open in 2024 following municipal approval [34, 35].

Despite accelerated momentum toward OPC implementation in several jurisdictions in the USA, there remains a paucity of literature characterizing and comparing successful OPC advocacy approaches in the USA. Studies of OPC advocacy in Canada [36–40], Australia [41, 42], and France [43] have generated vital insights on (in)effective approaches for building political and public consensus around OPC implementation. Insights gleaned from this scholarship include the salience of coalition-building in efforts to legalize OPCs [39, 41], the insufficiency of scientific evidence to effectively communicate OPCs’ viability to various audiences [36, 38, 40], and alignment with powerbrokers (especially law enforcement) as instrumental to localized OPC sanctioning [37]. Of note, much of the extant OPC advocacy literature has focused on efforts to sustain or scale-up OPCs in countries where these interventions have been piloted or fully implemented in key jurisdictions. Given the relative novelty of locally sanctioned OPCs in the USA (with NYC being the only municipality with sanctioned OPCs operational at the time of this writing), there is a critical need to document OPC advocacy approaches in contexts where legal precedents for authorization are non-existent, and local models of OPC implementation are sparse.

The USA is also governed by a unique patchwork of federal, state, and municipal laws [22], posing distinct challenges to introducing and implementing OPCs at scale. Efforts to legalize and implement OPCs have met success in only a handful of jurisdictions in the USA, even when jurisdictions employ similar advocacy strategies and pursue similar policy avenues to authorize these life-saving interventions [25, 31]. It is, therefore, imperative to explore the perceived value of OPC advocacy strategies in their specific implementation contexts in the USA, where municipal heterogeneities in social, political, and legal environments—coupled with geographic variations in the epidemiologic profiles of substance use and overdose [44, 45]—may attenuate the potency and viability of advocacy approaches with demonstrated success in other contexts, including within the USA. Accordingly, we conducted a qualitative study in multiple localities to glean stakeholder perspectives on contextually relevant strategies for mobilizing political and public support for OPC authorization and implementation in the USA.

## Methods

### Design and procedures

**Fig. 1:**
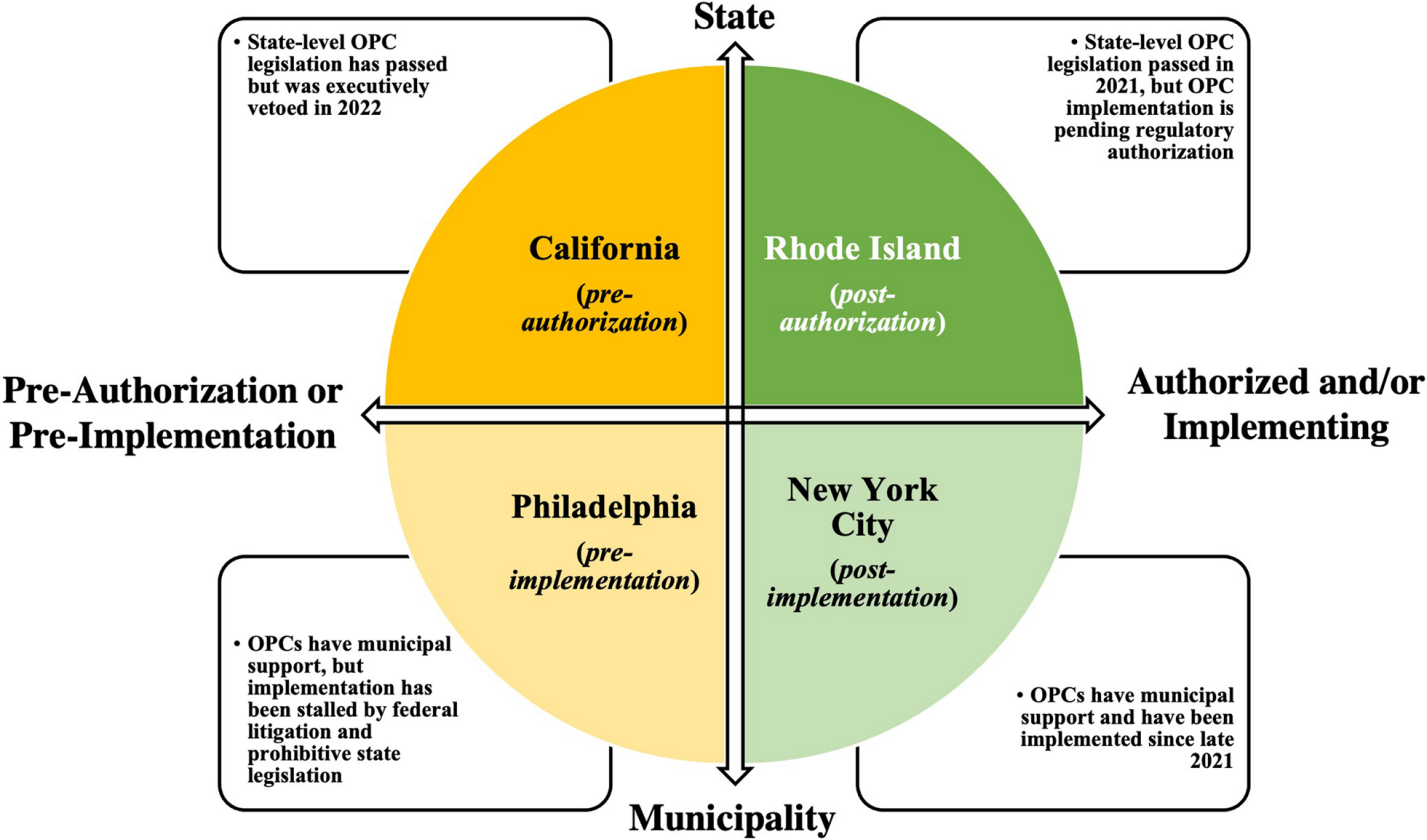
Purposively selected study sites, by overdose prevention center authorization and implementation status

We began by identifying individuals involved in OPC authorization and/or implementation efforts in four jurisdictions: California, NYC, Philadelphia, and Rhode Island. These jurisdictions were purposively selected to provide a representative and heterogeneous sample of OPC authorization and/or implementation experiences in the USA, as illustrated in Fig. 1, as well as to compare OPC advocacy at municipal (i.e., NYC, Philadelphia) and state (i.e., California, Rhode Island) levels. Two of these jurisdictions, Philadelphia and NYC, represent localities where efforts to introduce OPCs have focused on municipal authorization, although at the time of this writing, OPCs are operating only in NYC. In Philadelphia, efforts by the community-based organization Safehouse to open OPCs in two neighborhoods were stalled by strong opposition to proposed locations for OPCs, COVID-19 shutdowns, and protracted legal action, specifically federal litigation and the recent passing of Senate Bill 156, which explicitly bans OPC operation throughout Pennsylvania [23, 46]. California and Rhode Island, by comparison, are examples of states where legislative attempts to introduce OPCs have been successful, with Rhode Island successfully passing statewide legislation (2021-H5245A, 2021-S0016B) authorizing a two-year OPC pilot [34, 35]. While the California state assembly passed OPC legislation, Senate Bill 57, the bill was vetoed by Governor Gavin Newsom in August 2022 [47]. Shortly thereafter, a locally sanctioned, albeit temporary, OPC in the city of San Francisco, the Tenderloin Linkage Center, closed its doors on December 4, 2022, following substantial community backlash since the site’s opening earlier that year and dwindling support from Mayor London Breed’s administration [48].

Within each jurisdiction, we leveraged academic and community contacts to support recruitment of three to six stakeholders per location, which was theorized a priori to generate sufficient information for thematic saturation in the stratified sample; the study team reviewed interview debriefs and discussed emerging findings at least monthly to assess thematic saturation [49]. To generate information-rich insights into contextually appropriate and successful OPC advocacy strategies, we purposively recruited individuals intimately involved with OPC advocacy, authorization, and/or implementation in the four study jurisdictions, including lawmakers co-sponsoring OPC-focused legislation, OPC policy analysts, community organizers, service providers from OPCs and harm reduction organizations, and researchers. Leveraging professional networks within the study team and those of our research and practice partners, we first generated exhaustive lists of potential OPC stakeholders to approach within each jurisdiction, seeking variation in occupation. We contacted nominated stakeholders via email, provided a brief explanation of study objectives, and invited them to participate in the study. Those interested in participating connected face-to-face, by telephone, or by video-conferencing with trained qualitative interviewers (JGR, JT, JNP), who described the study objectives and procedures in detail.

After providing verbal informed consent, participants then completed a 40–60-min in-depth interview aided by a semi-structured guide, broaching the following topics: coalition-building and stakeholder engagement strategies to promote OPC advocacy, authorization, and legislative activities; strategies for increasing OPC awareness and support among elected officials, law enforcement, media, and members of the public; communication approaches for promoting OPC advocacy and implementation; and employed strategies for responding to institutions, coalitions, or constituents opposing OPC authorization and/or implementation. Prior to the interview, participants also completed a brief (approximately two-minute) interviewer-administered structured survey documenting socio-demographics, professional affiliation, occupational history, and lived substance use experiences. Participants were offered a $25 gift card as compensation for their time. At the conclusion of the interview, interviewers elicited contact information for other relevant stakeholders, whom interviewers contacted and approached for interviews until new insights no longer emerged in subsequent interviews within each jurisdiction [49]. Interviews were audio-recorded, professionally transcribed, and quality checked by interviewers for comprehension and fidelity to the recorded dialogue.

### Analysis

Guided by the tenets of inductive thematic analysis [50] and multi-cycle coding [51], four members of the study team (JGR, ET, JT, JNP) began by reading each transcript, line-by-line, and summarized salient concepts present in the texts (open coding). Emerging concepts were consolidated and transformed into standalone codes (focused coding), which were iteratively refined by the first author (JGR) through continuous transcript review, memo-writing, and discussion among coders. We then piloted the preliminary codebook by manually applying codes to one transcript from each jurisdiction, which facilitated assessment of code clarity and comprehensiveness. After revising and finalizing the codebook, the first author hierarchically embedded all codes into discrete overarching thematic domain, or parent codes, articulated in the semi-structured interview guide (axial coding) and imported the codebook into ATLAS.ti version 9 (Scientific Software Development GmbH, Berlin, Germany) for line-by-line coding and textual analysis.

The first author then applied sub-codes, nested within parent codes, to text segments within each transcript. After coding all transcripts, we exported and charted coded text segments by jurisdiction to first identify OPC authorization/implementation conditions and advocacy approaches deemed appropriate, successful, or relevant *within* each locality. Moving from within-case to across-case analysis [52], we then inspected emerging insights from each jurisdiction, identifying shared attributes between them and revising axial codes, as appropriate. Analytic memo-writing and ongoing discussion among the study team facilitated continued exploration, refinement, and crystallization of salient themes within each locality as well as textual data patterns across localities and stakeholder attributes (i.e., occupation), enhancing credibility and confirmability of thematic insights emerging from interview transcripts [53].

## Results

A total of 17 stakeholders across jurisdictions were identified purposively (*n* = 12) or via snowball sampling (*n* = 5) and completed in-depth interviews. Table 1 presents characteristics of the participants from Rhode Island (*n* = 6), California (*n* = 4), NYC (*n* = 4), and Philadelphia (*n* = 3) interviewed between July 2022 and February 2023. The median age was 54 years (interquartile range [IQR] 42–62 years). Half of participants were cisgender women (*n* = 9), and most were non-Hispanic white (*n* = 15). The most represented occupational groups were service providers (*n* = 6) and advocates/organizers (*n* = 6), followed by lawmakers/government officials (*n* = 3) and academic researchers (*n* = 2). Participants had extensive experience in OPC advocacy, reporting a median of 5 years of experience (IQR 4–15 years). Three participants self-reported lived experience with substance use.

**Table 1:** Descriptive sample statistics of interviewed stakeholders (*N* = 17)

Characteristics	Number (*n*)	Percent (%)
Jurisdiction		
Rhode Island	6	35.3
California	4	23.5
New York City	4	23.5
Philadelphia	3	17.7
Interview modality		
Virtual	11	64.7
Face-to-face	6	35.3
Age, in years (*median*, *IQR*)	54	42–62
Gender		
Cisgender woman	9	52.9
Cisgender man	7	41.2
Non-binary/genderqueer	1	5.9
Race and ethnicity		
Non-Hispanic white	15	88.2
Non-Hispanic black	1	5.9
Hispanic/Latinx (any race)	1	5.9
Occupation		
Service provider	6	35.3
Advocate/organizer	6	35.3
Lawmaker/government official	3	17.7
Academic researcher	2	11.7
Professional experience, in years (*median*, *IQR*)	5	4–15
Lived substance use experience	3	17.7

*IQR* Interquartile range

In what follows, we present the advocacy strategies that participants perceived as most valuable in increasing support for OPC authorization and/or implementation across jurisdictions. These advocacy strategies clustered around five overarching domains, including: (1) embedding OPC advocacy into broader overdose coalitions; (2) building rapport with a plurality of powerbrokers; (3) tailoring OPC communications to different audiences in different contexts; (4) challenging OPC opposition and misinformation; and (5) fostering public trust through transparency in OPC decision-making and implementation.

### Embedding OPC advocacy into broader overdose coalitions

Participants discussed successes fostering support for OPCs by embedding their advocacy into broader dialogs and efforts related drug overdose, including multi-sectoral policymaking coalitions. Across jurisdictions, multi-sectoral coalitions like the Mayor’s Commission on Opioid Use Disorder (Philadelphia), the Alliance for Saving Lives (California), and the Substance Use Policy, Education and Recovery Political Action Campaign (Rhode Island) were characterized as critical vehicles for advocates to shape high-level policy dialogs on appropriate solutions to surging overdose deaths—building momentum for OPC authorization and implementation planning efforts. This service provider, for example, argued that maintaining an early, consistent presence and sustaining representation in the Mayor’s Commission on Opioid Use Disorder was essential to communicating the benefits of OPCs to various stakeholders represented in a multi-sectoral coalition, which helped catalyze OPC planning in Philadelphia:

We formed the Overdose Prevention Task Force, then it expanded to the Mayor’s Commission on Opioid Use Disorder. That’s where overdose prevention sites in Philadelphia really began. It became a loud and open public discussion about what the possibilities were of having one [an OPC] in Philadelphia as a tool to prevent overdoses...As we began internal discussions, we quickly realized that we needed to broaden the stakeholder net, and we did that by inviting people from larger systems...We expanded it to fire, police, emergency, and the health department. We began to have different stakeholders come in and talk about overdoses in Philadelphia.

Alignment with these broader coalitions oftentimes did not always yield immediate OPC policy gains, but participants argued that prolonged engagement with these coalitions was essential to cultivating demand for new approaches to overdose prevention. Stakeholders explained how this prolonged engagement served to sensitize policymakers and the public to OPCs as viable policy solutions to the overdose crisis. As this Rhode Island advocate states:

You have to start somewhere, even if it's just a couple of years of having the conversation and raising awareness of the situation and that there are other non-traditional avenues people are taking in lieu of reverting to the things we’ve been doing for years as the [overdose death] numbers continue rising...Even if there's no instant gratification, and you have some hard conversations, you have to start somewhere...Know that you're going to be in it for the long haul.

### Building rapport with a plurality of powerbrokers

Participants emphasized the importance of rapport-building with stakeholders, whether they be lawmakers or other government officials, in advancing the OPC authorization and implementation agendas. Nowhere was this more evident than in Rhode Island, where participants described prolonged facetime with legislators as a key tenet of their advocacy strategy. As this service provider recalled, sustained communication and relationship-building with policymakers was essential to increasing visibility of the OPC movement and making OPC authorization a legislative priority:

I was advocating at the Statehouse, along with a few really dedicated folks...who were there frequently talking to lawmakers about harm reduction centers [OPCs]. I think that presence was important...If you want to change the law, you must talk to lawmakers...If you don’t have a one-on-one, face-to-face with a lawmaker, you’re not going to change the law.

Moving from OPC legislation (as in Rhode Island) to implementation (as in NYC), NYC stakeholders similarly credited the success of their OPC advocacy activities, in part, to the relationships they forged with high-profile powerbrokers, like city health department and law enforcement leadership. OPC advocates could effectively position themselves—and their work—as solutions to the overlapping challenges of public drug use and fatal overdoses, which law enforcement elicited their support in addressing. Reflecting upon a pattern of law enforcement-facilitated referrals of PWUD to OnPoint preceding the public unveiling of its sanctioned OPCs, this advocate explains:

We were having discussions with law enforcement quite a bit around ‘benzo alley.’ People were out in the streets, laying down all day. It was very visible. I remember the Department of Health was really contemplating action along with the NYC Police Department. They came to talk to us [OnPoint] as an organization that could work with the population [PWUD], get them off the streets, and bring them in somewhere. That's something that we do...The NYC Police Department refers people to OnPoint. That was happening before the opening of the OPC. There was already a relationship with law enforcement. I think, in terms of implementation, that’s a really big thing.

Likewise, in the absence of a formal authorization letter from the mayor or police department, this NYC service provider described how forging and maintaining partnerships with the city health department was imperative to redirecting anticipated public opposition to the unveiling of NYC OPCs away from the implementing partner (OnPoint) to government entities, who could shield service providers from liability and other potential sources of social or legal interference to OPC implementation.

We had to have the strong backing of the health department. One thing that we asked them to do was run interference in the public sphere...We needed to focus on making sure that the launch and execution of the OPCs was flawless because we were about to be under incredible scrutiny...It was very important that there be a firewall between the community and the provider [OnPoint]. If the community was going to be upset about there not being a consultation, they would blame the health department, not us [OnPoint]. Very graciously, the health department agreed to this request and understood strategically why it was important that we were seen to have clean hands. The health department did that. They went to every community board meeting and every police precinct meeting for months.

### Tailoring OPC communications to different audiences in different contexts

Participant narratives revealed the importance of tailoring OPC advocacy communications to different audiences as well as to the distinct political context and overdose profile of each jurisdiction. In NYC, for example, participants recounted concerted efforts to emphasize how supervised drug consumption services were just one of many services OPCs offer. One NYC advocate explained how communications centering the healthcare and wraparound services OnPoint offered (in addition to supervised drug consumption) successfully diffused some public reticence toward OPC implementation:

If you instead say, ‘In the middle of East Harlem, there is a healthcare center that offers holistic health services, five meals a day, coffee, community, prescription services, [and] low threshold care—free to the entire community.’ If you said all of that, people would say, ‘That's amazing. That's something that usually exists in wealthy communities, not in ours.’

By comparison, OPC advocacy communications leveraging substance use disorder treatment and recovery narratives were deemed most effective at neutralizing oppositional voices to OPCs in Rhode Island. These communications specifically emphasized how OPCs can facilitate drug treatment engagement by reducing fatal overdose risks, especially in the context of a deadly fentanyl overdose crisis. As one lawmaker quipped, “*You can’t recover if you’re not alive.*” These types of messages, in contrast, were considered less palatable in settings like California, where the ubiquity of fentanyl in the illicit drug supply materialized more recently compared to eastern jurisdictions [54, 55]. As this service provider explains, California lawmakers and constituents were more receptive to messages amplifying OPCs’ potential to address public ‘nuisances’ related to drug use (i.e., syringe litter, outdoor drug use), helping to diffuse NIMBYism directed at OPCs:

There’s a generalized environment of frustration and discontentment around the visible poverty and drug use...People want real solutions that are going to ameliorate the harms that they are experiencing or seeing or reading in the media...Bringing drug use inside...is a very popular and easy talking point.

Moreover, efforts to characterize OPCs as cost-effective, pragmatic solutions to the overdose crisis resonated most profoundly with powerbrokers, specifically lawmakers and law enforcement officials, in various jurisdictions. Philadelphia and NYC stakeholders specifically lauded the success of communications underscoring how OPCs would dramatically reduce healthcare-related costs of reversing overdoses, including dispatching emergency medical services (EMS) and providing care in hospital emergency departments. Describing early dialogs with city officials regarding the feasibility of implementing OPCs in Philadelphia, one service provider explained:

Having an EMS unit come over and over again, without a coordinated response, doesn’t make sense...They [OPCs] also are staffed with social workers to offer wraparound services. They also have a quick range to be able to provide medically-assisted treatment services. All of that is cost-effective. We were selling it as, ‘This is going to cost you less money if we begin to coordinate it.’

As this advocate shared, messages emphasizing EMS diversion attributed to OPC implementation resonated profoundly with NYC government officials, especially in the wake of the COVID-19 pandemic:

In the last three years, we had a pandemic, a strained health care system...ambulances racing up and down the streets...You just knew people were dying, and ambulances couldn’t reach people quickly enough...Being able to say, ‘We avoided calling EMS 700 times in the last year,’ was a big deal.

### Challenging OPC opposition and misinformation

Across jurisdictions, participants described both organized and piecemeal opposition from various sources (e.g., law enforcement, constituents) to OPC advocacy efforts. Stakeholders emphasized that confronting oppositional voices, rather than ignoring them, was critical to galvanizing support for OPC authorization/implementation efforts. By reframing the overdose crisis as a public health issue that service providers, rather than the criminal-legal system, were best equipped to solve, this advocate explained how partnerships with drug treatment providers and centering their testimony in legislative sessions helped discredit law enforcement opposition to legislation authorizing OPCs in California:

In the legislative environment, we have often led with treatment providers saying, ‘This is how we get people into treatment, not mandating or arresting...As the treatment experts, harm reduction is an essential part of treatment...Supervised consumption is effective, and this is why we’re supportive of it.’ Having a treatment provider say, ‘Mandated treatment is ineffective,’ has helped to undermine the authority of law enforcement as being an expert on treatment.

Likewise, in Rhode Island, participants explained how centering testimony from frontline service providers, including emergency medicine physicians and nurses, helped reinforce the viability and legitimacy of OPCs to skeptical or reticent policymakers. This lawmaker explained the rationale for strategically mobilizing specific actors to testify in support of legislation authorizing OPCs in Rhode Island:

If you know that your political leadership and law enforcement leadership are opposed...circumventing the leadership and going to those who are actually on the street, whether it be a patrolman or a doctor in an emergency room, may be best...Get as much understanding by hearing directly from those who are on the street. Go to those in an emergency room or a patrolman rather than a police chief.

### Fostering public trust through transparency in OPC decision-making and implementation

Lastly, participants emphasized how galvanizing support for OPC authorization and implementation hinged on transparency of OPC advocacy activities. In Philadelphia, for example, participants discussed how law enforcement galvanized community opposition to OPCs by sharing implementation plans, shared confidentially by OPC advocates, prior to their public unveiling. As this advocate from Philadelphia recalled:

The police lieutenant went back to the neighbors and told them [of confidential plans to open an OPC]. Word got back to the neighbors, who said in the media they had a meeting with that cop. They learned on that day that this [OPC planning] was happening. We announced our plans because we always wanted to be transparent. We announced our plans at the Governor’s office. It was a media event, and we had a plan to have a community meeting a week later in that neighborhood because we weren’t trying to hit and run...The press conference was ugly. People were screaming at us. They were calling us sneaks.

Reinforcing the importance of public transparency in contexts where OPC planning is ongoing, this California advocate described his empathy for community members who felt ‘blindsided’ by disjointed communications about plans to open OPCs in these two Philadelphia neighborhoods, stating:

I think that by working with the folks in the community where a site might be located, you can address a lot of the concerns...I think it’s a mix of validating people’s concerns, showing the evidence, bringing them in, and helping them see that we’re all sharing the same goal of making the community safer.

In contrast, NYC participants asserted that inviting policymakers, media, and the public into their OPCs, rather than hosting large public forums like town halls or press events, was sufficient to neutralizing oppositional voices to OPC implementation. As this service provider explained:

The only thing I’ve ever found that works is bringing people in [to the OPC] and saying, ‘Be in the room’...Even for your most staunch detractor, you can neutralize dissent by allowing people into the space and spending time in the space. People cannot uphold those preconceptions and that stigma...after they spend time in the space.

NYC participants also explained how providing wraparound services in key neighborhoods for protracted time periods, predating the tacit authorization of OPCs in NYC, was essential to building community trust. Reflecting upon effective approaches for addressing public and political NIMBYism toward OPCs, this NYC service provider advised other jurisdictions to co-locate newly established OPCs within existing harm reduction interventions implemented by reputable and trusted organizations:

It’s going to be challenging if you’re a new service provider and if it’s a new space. We could not be NIMBY-ed. We’ve been in the area for 22 years…We’re a long-standing program. You couldn’t NIMBY us...Make yourself un-NIMBY-able.

## Discussion

Study findings underscore the perceived value of several strategies to amplify public and political support for OPCs, even in circumstances where OPC legislation had been recently vetoed by a governor (California), implementation plans were stalled by legal action (Philadelphia), or formal municipal authorization is still pending (NYC). While specific to the socio-political context and overdose profile of each locality, some common strategies were employed across jurisdictions to render OPCs more palatable to powerbrokers and the public while reinforcing their appropriateness and relevance in policymaking circles. For instance, participants consistently emphasized how entrenching themselves into broader multi-sectoral coalitions for overdose prevention was crucial to sustaining the momentum of their OPC advocacy activities. Participants explained how claiming ‘seats at the table’ in municipal and statewide opioid and overdose coalitions was critical for influencing policy dialogs and cultivating demand for OPCs. Consistent with findings from studies of OPC policymaking internationally [25, 40, 41], coalition-building is an essential tenet of OPC advocacy, even if these activities do not immediately generate policy gains. Moreover, this strategy is consistent with approaches framing OPCs as a single component of a broader, comprehensive continuum of interventions to address drug overdoses [40, 56], which have been employed successfully in OPC advocacy outside the USA, including in Australia [42] and Canada [36, 38], as well as broader harm reduction advocacy, like statewide syringe services programs authorization [57–59], within the USA.

Findings also underscore the importance of communications that are uniquely tailored to different audiences and resonate in the unique socio-political environments in which OPC advocacy efforts are occurring. For example, while effective in increasing support for OPC authorization in Rhode Island, messages linking OPCs to the goals of the treatment and recovery movements (e.g., OPCs opening pathways to substance use treatment services) were seen as less relevant in settings like NYC, where centering the wraparound services OPCs offered was perceived as most palatable to policymakers and constituents. Likewise, participants emphasized the importance of uniquely differentiating OPC advocacy messages to different audiences. Lawmakers and other government officials, for instance, were generally perceived as more receptive to messages highlighting OPCs as a pragmatic, cost-savings solution to the overdose crisis in their respective jurisdictions. By comparison, participants perceived members of the public to be more receptive to messages communicating OPCs’ alignment with broader social goals/values related to substance use (e.g., safe syringe disposal, bringing drug use indoors). Consistent with perspectives of harm reduction advocates in the USA [25, 60] and internationally [36, 61], successful OPC advocacy hinged upon communicating specific messages about OPCs, to specific audiences, in specific contexts. Communications could also helpfully differentiate messaging to audiences that are more susceptible to OPC-related misinformation, creating opportunities to educate and sensitize different audiences to the multiple benefits (beyond overdose prevention) and value of OPCs in the landscape of harm reduction approaches [29, 62].

To effectively discredit oppositional voices, participants further emphasized the need to center the voices of ‘credible’ stakeholder groups from the health sector (e.g., frontline emergency medicine physicians and nurses, substance use treatment providers) in their advocacy efforts. Rapport-building with a plurality of stakeholders—powerbrokers like lawmakers, health department officials, and law enforcement in particular—is a proven advocacy strategy for complementary harm reduction interventions, including decriminalization of syringe possession and drug policy reform [57, 63], both within and outside the USA. Jurisdictions pursuing OPC legalization at the state level will also need the support of elected officials like governors, who wield veto power to overturn bills passed by state legislatures, as observed in California—suggesting elected officials and other powerbrokers (e.g., city mayors, law enforcement leadership, health department officials) will have unequal influence in the OPC policymaking process. As French scholars have warned from the experience opening drug consumption rooms in Paris [43], OPC advocates in the USA should ensure other critical voices, like PWUD and their families, are not sidelined at the expense of centering the social capital and perceived credibility of other groups. Silencing people with lived experience, even inadvertently, in OPC advocacy may perpetuate substance use stigmas [25, 64, 65], re-marginalizing populations who stand most to benefit from OPC implementation.

Lastly, stakeholders described transparency as critical to building public trust in OPC authorization movements and implementation efforts. Reacting to the tense legal battle unfolding in Philadelphia over Safehouse [66], participants reaffirmed the importance of being the first of many voices unveiling major OPC-related legislative or authorization efforts. In NYC, where OnPoint rapidly mobilized services to open the nation’s first sanctioned OPCs ahead of protective statewide or federal legislation, participants emphasized the impetus to publicly showcase the OnPoint model, through multi-channel dissemination and site tours for journalists and lawmakers, to subvert misconceptions of OPCs and enhance the perceived legitimacy of OPCs. This type of transparency, however, may be challenging in localities that are unable to arrange visits to a nearby OPC and where extensive evidence from Canada, Australia, Europe, or even other jurisdictions in the USA can be dismissed by skeptics as contextually irrelevant [24, 67]. Adhering to recommendations stemming from OPC advocacy scholarship in Australia [41], Canada [39], and Europe [68], national coalitions could play an important role for OPC advocacy in other contexts, sharing best-practices for OPC organizing, lobbying, and implementation.

Our findings are subject to several limitations. First, we purposively sampled participants who were professionally involved in advocacy supportive of OPC authorization and implementation. Due to time and funding constraints, we did not elicit perspectives of other stakeholder groups opposing OPC-related legislation who might have offered alternative insights into the deployment and perceived value of specific OPC advocacy strategies. Second, our study focused principally on exploring advocacy strategies perceived as valuable by OPC stakeholders. We, therefore, did not explicitly interrogate advocacy strategies deemed invaluable or futile in specific contexts. Third, we relied exclusively on in-depth interviews to understand strategies for mobilizing public and political support for OPC authorization. Finally, we did not elicit perspectives on stakeholders from all jurisdictions in the USA attempting to authorize and implement OPCs (e.g., Maryland, Massachusetts, Seattle, Vermont), nor did we explicitly investigate approaches for OPC authorization and implementation amidst a complex and sometimes contradictory ecosystem of federal, state, and municipal legislation. The primary goal of this study was to understand advocacy strategies for galvanizing support from various audiences—providing a foundation for future scholarship on OPC authorization and implementation in the USA.

## Conclusions

Our findings provide a helpful roadmap for stakeholders in the USA to plan, structure, and sequence their advocacy activities to maximize their impact, despite OPC legal ambiguities at the federal level. First, jurisdictions should embed their advocacy in higher-level, multi-sectoral overdose response coalitions. Second, OPC implementers should establish and maintain partnerships with various powerbrokers, from lawmakers to health departments, to amplify the impact of their advocacy. Third, efforts should be made to develop tailored communications emphasizing specific benefits of OPCs to different audiences. Fourth, advocates should consider leveraging relationships with publicly ‘credible’ entities to support efforts to neutralize oppositional actors and the OPC-related misinformation they may be peddling. Lastly, cross-jurisdictional peer-to-peer exchanges of OPC advocates, through planned OPC site visits or other national meetings, could support wider dissemination and translation of successful approaches for OPC organizers in other jurisdictions in the USA. These lessons could build upon the monumental efforts made authorize and implement OPCs in the USA.

## Data Availability

To protect participant confidentiality, the data used in the present manuscript are not available for public dissemination.

## Abbreviations

IQR: Interquartile range
NYC: New York City
OPC: Overdose prevention center
USA: United States of America
